# Sex differences in the redox response to fine particulate matter (PM_2.5_) air pollution protects female mice against metabolic and cardiac injury

**DOI:** 10.14814/phy2.70536

**Published:** 2025-09-02

**Authors:** Michael Kitching, Amanda Ribble, Ashley Wright, Aruni Bhatnagar, Petra Haberzettl

**Affiliations:** ^1^ Center for Cardiometabolic Sciences, Christina Lee Brown Envirome Institute, Division of Environmental Medicine University of Louisville Louisville Kentucky USA

**Keywords:** air pollution, cardiovascular disease, female, fine particulate matter PM_2.5_, sex, type 2 diabetes

## Abstract

Fine particulate matter (PM_2.5_) exposure increases the cardiometabolic disease risk. While there is extensive research on how PM_2.5_ impairs cardiometabolic health in male mice, its health impact is largely unexplored in females. To examine PM_2.5_‐induced cardiometabolic effects in females, female and male mice (*n* = 10/group) on a regular (12 h:12 h, RLC) or disturbed (18 h:6 h, DLC) light–dark cycle were exposed to concentrated ambient PM_2.5_ (CAP) for 30 days. In females, CAP exposure neither impacted glucose tolerance nor skeletal muscle or liver insulin sensitivity. Western blot analysis of cardiac insulin signaling in females and males showed that CAP impaired insulin‐stimulated phospho‐Akt in the heart of male mice but did not impair cardiac insulin signaling in females. While CAP exposure increased circulating malondialdehyde (MDA) and decreased plasma nitrite (NO_x_) in male mice, females were protected against CAP‐induced systemic oxidative/nitrosative stress. Similarly, CAP exposure increased thiobarbituric acid reactive substances (TBARS) and depleted glutathione only in the male lungs. Interestingly, in females, CAP increased pulmonary oxidized‐glutathione (GSSG) without decreasing reduced glutathione (GSH) indicating advanced pulmonary antioxidant defense in female mice also supported by higher pulmonary antioxidant enzymes *mRNA* abundance. Our results show that female mice are protected against cardiometabolic PM_2.5_ toxicity possibly by preventing PM_2.5_‐induced pulmonary oxidative stress.

## Graphical abstract

Female mice are protected against metabolic and cardiac PM_2.5_ toxicity by a better protection against PM_2.5_‐induced pulmonary oxidative stress. This is supported by our findings that lung antioxidant enzyme mRNA expression is higher in female mice when compared to the males

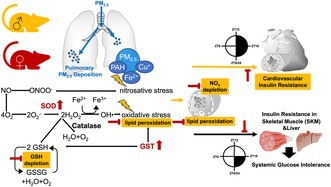

## 1 INTRODUCTION

Exposure to environmental air pollution is a strong contributor to premature mortality worldwide (WHO, 2024). As such, exposure to indoor and outdoor air pollution has been linked with 6.7 million premature deaths, 2.9 million in females and 3.8 million in males (Fuller et al., 2022). Although the higher association between air pollution exposure and premature mortality found in men indicates that males are more sensitive to polluted air than females (Fuller et al., 2022), research investigating sex‐specific effects of air pollution exposure on cardiovascular and metabolic health shows controversial results (Liao et al., 2023). Moreover, experimental studies on how sex impacts the responses to air pollution exposure are still limited, and while studies investigating female sensitivity to air pollution are expanding, a mechanistic understanding is still elusive.

Polluted air resulting from, for instance, industry, traffic, farming, mining, and wildfires contains a complex mixture of gaseous (i.e., ozone, nitric oxide, sulfur and nitrogen dioxide) and particulate matter (PM) components (Haberzettl et al., 2014). Particulate matter (PM) is characterized based on its size that limits its ability to enter and deposit in the body and determines PM toxicity. For instance, combustion‐derived ambient air pollution contains fine particulate matter (PM_2.5_, aerodynamic diameter ≤2.5 μm) that, when inhaled, can reach the lung alveoli and has been associated an increase in the incidence of cardiovascular disease (CVD) and type 2 diabetes (T2D) (Bhatnagar, 2022; Brook et al., 2013; Coogan et al., 2012; Haberzettl, O'Toole, et al., 2016; Humphrey et al., 2024; Klompmaker et al., 2024; Kramer et al., 2010; Pearson et al., 2010; Rajagopalan, Brook, et al., 2024; Rajagopalan & Landrigan, 2021; Sagheer et al., 2024; Sun et al., 2009). Although legislative actions such as the Clean Air Act helped to improve air quality, rising urbanization and an increase in the incidence and intensity of wildfires have started to reverse the air quality advances in Europe and the USA (Burke et al., 2023; Rajagopalan, Vergara‐Martel, et al., 2024; Stucki et al., 2023). Currently, the majority of the human population is exposed to PM_2.5_ levels above the World Health Organization recommended concentrations of 5 μg/m^3^ (WHO, 2021). Consequently, studies that identify susceptible populations and recognize sex‐specific sensitivities are crucial for personalized risk assessment. Moreover, identifying responses that are distinctive to one sex could help to identify the mechanism(s) by which PM_2.5_ increase the risk of developing CVD and T2D and consequently help to develop mechanism‐based sex‐specific intervention strategies. While there is growing evidence from epidemiological studies for a sex‐dependent sensitivity to PM_2.5_ (Liao et al., 2023), only a few experimental studies exist that test for the cardiovascular and metabolic PM_2.5_ toxicity in females. Two early studies by Qin et al. and Yan et al. examined cardiovascular and hepatic function after short‐term (4 weeks) PM_2.5_ treatment in female mice of different ages (Qin et al., 2018; Yan et al., 2020). These studies indicate an age‐dependent impact of PM_2.5_ on hepatic lipid metabolism and cardiac function possibly driven by the induction of oxidative stress. While these studies advance research in females, the experiments were only performed in female mice. One initial study that facilitated male and female mice indicates that PM exposure had a differential impact on pulmonary, systemic, and vascular responses (Wang et al., 2022). Although significant differences between male and female were very limited, this study suggests that female mice are more susceptible for PM_2.5_ exposure. In contrast, another study showed that female mice are protected against PM_2.5_‐induced systemic inflammation and oxidative stress as well as endothelial progenitor cell depletion (Liu et al., 2021). A more recent exposure study showed that female mice are more prone to develop systemic insulin resistance upon long‐term (24 weeks) PM_2.5_ exposure, which was accompanied by exacerbated lipid accumulation in the liver and a dysregulation of hormone release by the hypothalamus–pituitary–adrenal (HPA) axis (Li, Sun, et al., 2020). Similar to the epidemiological findings, the results of these experimental studies are controversial, and more research is needed to identify the sex‐specific mechanism(s) that drive the cardiovascular and metabolic toxicity of PM_2.5_.

Recent research has identified multiple environmental factors, lifestyle choices, and health conditions that increase the susceptibility to PM_2.5_ exposure (Rajagopalan, Vergara‐Martel, et al., 2024). For instance, the association between air pollution exposure and the incidence of T2D increased with adiposity in a human cohort (Li et al., 2021). We and others have shown that exposure to concentrated ambient PM_2.5_ (CAP) exacerbates glucose intolerance and insulin resistance in mice that were either fed with a high‐fat diet (Haberzettl, O'Toole, et al., 2016; Sun et al., 2009) or exposed to an extended light cycle (Ribble et al., 2023). Epidemiological evidence also indicates that exposure to air pollution increases the CVD risk and CVD progression in individuals with circadian syndrome (Liu et al., 2024)—a pathological state that extends the metabolic syndrome with circadian disturbances, for example, shortened or extended sleep duration (Zimmet et al., 2019). On the other hand, it has been shown that PM_2.5_ exposure increases the risk of developing the circadian syndrome in humans (Hu et al., 2023) and that exposure to PM_2.5_ impacts the circadian rhythm in metabolically active organs such as the liver and adipose tissue in mice (Li, Wang, et al., 2020; Palanivel et al., 2020; Rajagopalan et al., 2020). While 30 weeks of concentrated PM_2.5_ exposure have been found to impair day‐ and night‐time energy expenditure and increase plasma corticosterone levels (Palanivel et al., 2020), results obtained from our previous study (Ribble et al., 2023) indicate that a shorter exposure (30 days) does not interfere with the central regulation of the circadian rhythm as neither metabolic parameters nor corticosterone levels are impacted. However, changing the light cycle shifted the activity pattern and changed plasma corticosterone levels. Taken together, epidemiological and experimental evidence suggests that circadian disturbances increase the susceptibility to PM_2.5_ and that vice versa PM_2.5_ exposure alters circadian rhythmicity consequently increasing the risk of developing the circadian syndrome, T2D, and CVD as well as their progression and severity. However, it is unclear whether males and females are impacted differently. Our previous studies showed that in male mice disturbed in their circadian rhythm, CAP exposure for 30 days exacerbated glucose intolerance and insulin resistance induced by the extended light exposure (Ribble et al., 2023). We have also demonstrated that, in male mice, an exposure to CAP for either 9 or 30 days induces cardiac and aortic insulin resistance independent of high‐fat diet feeding or extended light exposure (Haberzettl, McCracken, et al., 2016; Haberzettl, O'Toole, et al., 2016; Hill et al., 2021). However, it is unclear whether and how inhalation of PM_2.5_ impacts cardiometabolic health in female mice. Thus, we investigated the effects of PM_2.5_ exposure on systemic glucose homeostasis and tissue‐specific insulin sensitivity in female mice kept on a regular or disturbed light cycle (DLC). In contrast to our observations in male mice (Ribble et al., 2023), results from the current study suggest that female mice are protected against the cardiometabolic impact of PM_2.5_ exposure.

## 2 MATERIALS AND METHODS

### 2.1 Animal experiments

**FIGURE 1:**
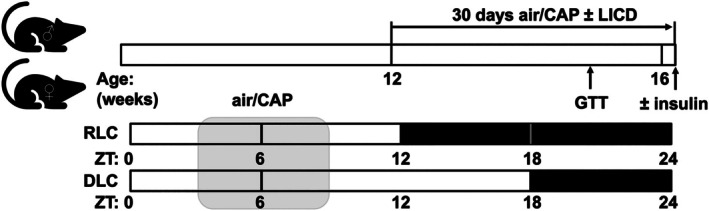
Experimental protocol. Male and female 12‐week‐old mice either maintained on a regular cycle (RLC, 12 h light: 12 h dark) or switched to a disturbed light cycle (DLC, 18 h light: 6 h dark, light‐induced circadian dyssynchrony, LICD) were exposed to concentrated ambient fine particulate matter (CAP) or inhaled HEPA‐filtered air for 30 consecutive days (6 h per day, Zietgeber time, ZT3‐9). Glucose tolerance test (GTT) was performed at day 21 of the experimental protocol. Finally, mice were injected with either saline (control) or insulin to assess tissue‐specific insulin sensitivity.

All animals were handled in accordance with the American Physiological Society (APS) Guiding Principles in the Care and Use of Animals following protocols approved by the University of Louisville Institutional Animal Care and Use Committee (IACUC, no. 24448). Except for experiments that require fasting, mice were provided with food and water ad libitum. Male and female mice on a C57/Bl6 background were maintained on a standard light: dark cycle (12 h [ZT0‐ZT12] light: 12 h [ZT12‐ZT24] dark, ZT, Zeitgeber time; ZT0 = beginning of light cycle at 6:00 am) until 12 weeks of age. Mice (*n* = 10 male and female mice per group, injections with saline, *n* = 5 or insulin, *n* = 5) were then randomly assigned to the different treatment groups. To disturb the circadian rhythm (Ribble et al., 2023), one group of either male or female mice was switched to a disturbed light cycle (DLC, 18 h [ZT0‐ZT18] light: 6 h [ZT18‐ZT24] dark) whereas the control groups were kept on the regular light cycle (RLC, 12 h [ZT0‐ZT12] light: 12 h [ZT12‐24] dark). Mice either maintained on RLC or switched to a DLC were exposed to concentrated ambient fine particulate matter (CAP) or inhaled HEPA filtered air for 30 days for 6 h per day (ZT3‐9, Figure 1). As detailed previously (Ribble et al., 2023), CAP is concentrated from the ambient air using a Versatile Aerosol Concentration Enrichment System (VACES) and mice were exposed to environmentally relevant concentrations of PM_2.5_ ranging from 56 to 100 μg/m^3^. Body weights were measured weekly. At day 21, a glucose tolerance test (GTT) was performed as described (Haberzettl, O'Toole, et al., 2016; Ribble et al., 2023). For this, mice were fasted for 6 h. After measuring basal glucose levels, mice were interperitoneally injected with 1 g glucose in sterile saline per kg body weight. Blood glucose levels were measured at 0, 5, 15, 30, 60, and 120 min (Accu‐Check, Roche, United States). Following 30 days of the experimental protocol, fasted (6 h) mice were interperitoneally injected with insulin (Humulin‐RP, Eli‐Lilly, United States, 1.5 U/kg body weight) or saline (control) to assess tissue‐specific insulin sensitivity. Tissues were harvested around ZT3 within 15 min after injection and snap frozen for biochemical analysis.

### 2.2 Western blot analysis

Insulin sensitivity in skeletal muscle (SKM), liver, and hearts was determined by Western blot as described before (Haberzettl, O'Toole, et al., 2016; Ribble et al., 2023) using the following antibodies: phospho‐Akt (Ser473, Cat no.: 9271, Cell Signaling, United States, 1:1000), Akt (Cat no.: 9272, Cell Signaling, United States, 1:1000), and anti‐rabbit IgG secondary antibody (Cat no.: 7074, Cell Signaling, United States 1:2000).

### 2.3 Biochemical analysis of redox changes

To assess systemic redox changes, plasma levels of malondialdehyde (MDA) and the nitric oxide breakdown product nitrite (NO_x_) were measured using commercially available kits (Lipid Peroxidation (MDA) Colorimetric Assay Kit, United States, ab118970; Griess assay, Promega, United States G2930). While MDA, a lipoperoxidation product, is commonly used as a marker for oxidative stress, the abundance of the nitric oxide breakdown product nitrite (NO_x_) is used as a marker for nitrosative stress (Cipak Gasparovic et al., 2017; Cordiano et al., 2023). To measure pulmonary redox changes, we used commercially available kits to detect either thiobarbituric acid reactive substances (TBARS, ZeptoMetrix, United States TBARS Assay Kit, 0801192) or reduced (GSH) and oxidized (GSSG) glutathione (Glutathione Colorimetric Detection Kit, United States, no. EIAGSHC) in the collected lung tissue. While the TBARS assay measures lipid peroxidation (mainly MDA), the GSH assay detects the conversion of reduced glutathione (GSH) to oxidized glutathione (GSSG) consequently decreasing the levels of GSH while increasing GSSG levels thereby changing the ratio of GSH: GSSG (Aguilar Diaz De Leon & Borges, 2020; Jones, 2002). To avoid interferences with insulin signaling, only samples of saline‐injected mice were used to detect redox changes.

### 2.4 Quantitative real‐time (qRT)‐PCR

Tissues (lungs and epididymal white adipose tissue, eWAT) collected from saline‐injected mice were used to perform quantitative real‐time (*qRT*)‐PCR following the previously described protocols (Haberzettl, O'Toole, et al., 2016; Ribble et al., 2023). In the collected adipose tissue of saline‐injected air or CAP‐inhaling female mice kept on an RLC or switched to a DLC, the abundance of the pro‐inflammatory *mRNAs tumor necrosis factor α* (*Tnf‐α*), *interleukin 1β* and *6* (*Il1β*, *Il6*), *monocyte chemoattractant protein 1* (*Mcp1* aka *chemokine* (*C‐C motif*) *ligand 2*, *Ccl2*), and *macrophage inflammatory protein‐1‐α* (*Mip1‐α*, aka *Chemokine* (*C‐C motif*) *ligand 3*, *Ccl3*) were measured by (*qRT*)*‐PCR*. The lung tissue of saline‐injected air‐inhaling male and female mice kept on an RLC was used to measure the *mRNA* abundance of the antioxidant enzymes *superoxide dismutase* (*Sod1*, *2*, and *3*), *glutathione transferase* (*Gsta*, *m*, and *p*), *catalase* (*Cat*), and *heme oxygenase‐1* (*Hmox1*) and their transcription factor *nuclear factor‐erythroid factor 2‐related factor 2* (*Nrf2*) by *qRT*‐PCR.

### 2.5 Statistical analysis

For statistical analysis, Graph Pad Prism V 9.0 software was used. Experimental data are presented as mean ± standard error (SE). To determine statistical significance (*p* < 0.05), an unpaired Student's *t*‐test was performed for two‐group comparisons between air and CAP or RLC and DLC as indicated in the respective legends. A two‐way ANOVA followed by Tukey post hoc test was used to compare multiple groups.

## 3 RESULTS

### 3.1 Exposure to PM_2.5_ does not impact glucose homeostasis in female mice

**FIGURE 2:**
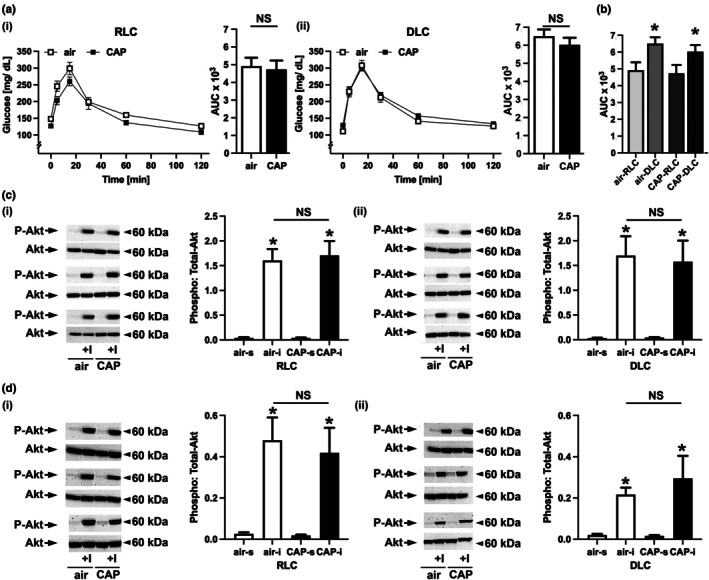
Glucose tolerance and skeletal muscle and liver insulin sensitivity in female mice. (a) Blood glucose levels during glucose tolerance test (GTT) and total glucose excursion calculated by integrating the area under the curve (AUC) for female mice that were maintained on a regular light cycle (RLC, i) or switched to a disturbed light cycle (DLC, ii) that inhaled air or concentrated ambient fine particulate matter (CAP). Data are presented as mean ± SE. For statistical comparisons of the calculated AUC between air and CAP inhaling mice that were kept either on an RLC (NS = not significant; RLC‐air vs. RLC‐CAP) or DLC (NS = not significant; DLC‐air vs. DLC‐CAP) an unpaired two‐tailed student's *t*‐test was used. (b) To compare total glucose excursion (AUC) between female mice kept on RLC or DLC that are exposed to either air or CAP, an unpaired two‐tailed student's *t*‐test was used (^*^ *p* < 0.05; RLC‐air vs. DLC‐air; RLC‐CAP vs. DLC‐CAP). Representative Western blots and quantitative analysis of the insulin‐stimulated Akt phosphorylation in (c) skeletal muscle (SKM) and (d) liver of female mice kept on an RLC (i) or switched to a DLC (ii) inhaling air or CAP for 30 days. Mice were injected with either saline or insulin (+I, 1.5 U/kg, 15 min). Data are presented as mean ± SE, *n* = 5 per group. For statistical analysis of the Western blot data, a two‐way analysis of variance (ANOVA) followed by a Tukey's multiple comparisons test was used. Statistical significance between saline and insulin injected mice that inhaled air or CAP is indicated by * (*p* < 0.05, air vs. air‐I, CAP vs. CAP‐I) for mice kept either on an RLC or DLC. No significant differences in the insulin‐stimulated phosphorylation of Akt between air and CAP inhaling mice kept either on an RLC or DLC are indicated by NS (not significant; RLC: air‐I vs. CAP‐I; DLC: air‐I vs. CAP‐I).

To test whether CAP exposure impacts glucose homeostasis in female mice, we performed a glucose tolerance test (GTT) in female mice kept on a regular (RLC) or switched to a disturbed (DLC) light cycle that inhaled air or CAP for 21 days (Figure 2a). We found that CAP exposure neither altered glucose extrusion in female mice kept on an RLC (Figure 2ai) nor changed glucose removal in female mice kept on a DLC (Figure 2aii). Additionally, fasting blood glucose and plasma insulin levels (Table 1) were similar between female mice inhaling air or CAP either kept on an RLC or DLC (glucose [mg/dL] RLC‐air: 131 ± 5, RLC‐CAP: 135 ± 4, *p* = 0.569, DLC‐air: 137 ± 7, DLC‐CAP: 140 ± 5, *p* = 0.767; insulin [ng/mL] RLC‐air: 0.39 ± 0.01, RLC‐CAP: 0.38 ± 0.01, *p* = 0.790, DLC‐air: 0.38 ± 0.01, DLC‐CAP: 0.39 ± 0.01, *p* = 0.265). While female mice kept on a DLC were protected from the impact of CAP on glucose homeostasis, the female sex did not protect against glucose intolerance that resulted from the maintenance on a disturbed light cycle (Figure 2b). Extending the light cycle significantly increased the calculated area under the curve (AUC) during GTT in both female mice that inhaled either air or CAP (Figure 2b).

**TABLE 1:** Physiological parameter in female mice.

	RLC			DLC		
	air	CAP	*p*	air	CAP	*p*
BW (g)	21.1 ± 0.4	20.8 ± 0.4	0.542	22.0 ± 0.5	22.0 ± 0.7	0.990
Lung (% BW)	0.69 ± 0.02	0.67 ± 0.02	0.412	0.69 ± 0.02	0.64 ± 0.02	0.086
Heart (% BW)	0.55 ± 0.03	0.55 ± 0.02	0.978	0.57 ± 0.02	0.53 ± 0.02	0.372
Liver (% BW)	4.27 ± 0.09	4.19 ± 0.12	0.598	3.86 ± 0.18	4.36 ± 0.06	0.616
Spleen (% BW)	0.41 ± 0.01	0.43 ± 0.02	0.184	0.44 ± 0.02	0.42 ± 0.01	0.348
Muscle (%BW)	1.44 ± 0.06	1.43 ± 0.05	0.910	1.52 ± 0.04	1.44 ± 0.06	0.271
Kidney (%BW)	1.29 ± 0.02	1.33 ± 0.04	0.476	1.34 ± 0.05	1.28 ± 0.02	0.281
Glucose (mg/dL)	131 ± 5	135 ± 4	0.569	137 ± 7	140 ± 5	0.767
Insulin (ng/mL)	0.39 ± 0.01	0.38 ± 0.01	0.790	0.38 ± 0.01	0.39 ± 0.01	0.265

*Note*: Body weight (BW), tissue: body weight ratio (in %), as well as fasting blood glucose and plasma insulin levels measured in female mice kept on a regular light cycle (RLC) or switched to a disturbed light cycle (DLC) that inhaled either air or concentrated ambient fine particulate matter (CAP) for 30 days. Data are mean ± SE; *n* = 10; for plasma insulin: *n* = 5. An unpaired Student's *t*‐test was used to compare the means of air or CAP inhaling mice kept either on an RLC or DLC.

As deficiencies in systemic glucose handling can be attributed to tissue‐specific insulin resistance, we tested for changes in insulin signaling in skeletal muscle (SKM) and liver. Intact insulin signaling in the SKM and liver is essential for the regulation of glucose homeostasis. In the SKM, insulin facilitates the uptake of glucose and consequently its removal from the circulation via the phosphorylation of Akt (da Silva Rosa et al., 2020; Kubota et al., 2011). In the liver, insulin inhibits gluconeogenesis by stimulating Akt phosphorylation which decreases the expression of gluconeogenesis proteins (Hatting et al., 2018; Titchenell et al., 2017). As female mice are protected against the impact of CAP on systemic glucose handling, we expected that insulin signaling in SKM and liver is unaffected by CAP as well. To test for intact insulin signaling, we measured the insulin‐stimulated phosphorylation of Akt in the SKM (Figure 2c) and liver (Figure 2d) of air or CAP inhaling female mice maintained on a regular (RLC, Figure 2c,di) or disturbed (DLC, Figure 2c,dii) light cycle by Western blot. In all groups, insulin injection induced the significant phosphorylation of Akt in both SKM and liver, and no significant differences were observed between the air and CAP groups. As expected, exposure to CAP did not impair the insulin‐stimulated phosphorylation of Akt in either SKM or liver of female mice maintained on an RLC or DLC.

Taken together, our results show that female mice kept on a disturbed light cycle are protected against the effects of CAP exposure on glucose tolerance (Figure 2aii) and SKM (Figure 2cii) or liver (Figure 2dii) insulin sensitivity.

### 3.2 Exposure to PM_2.5_, adiposity, and adipose tissue inflammation in female mice

**FIGURE 3:**
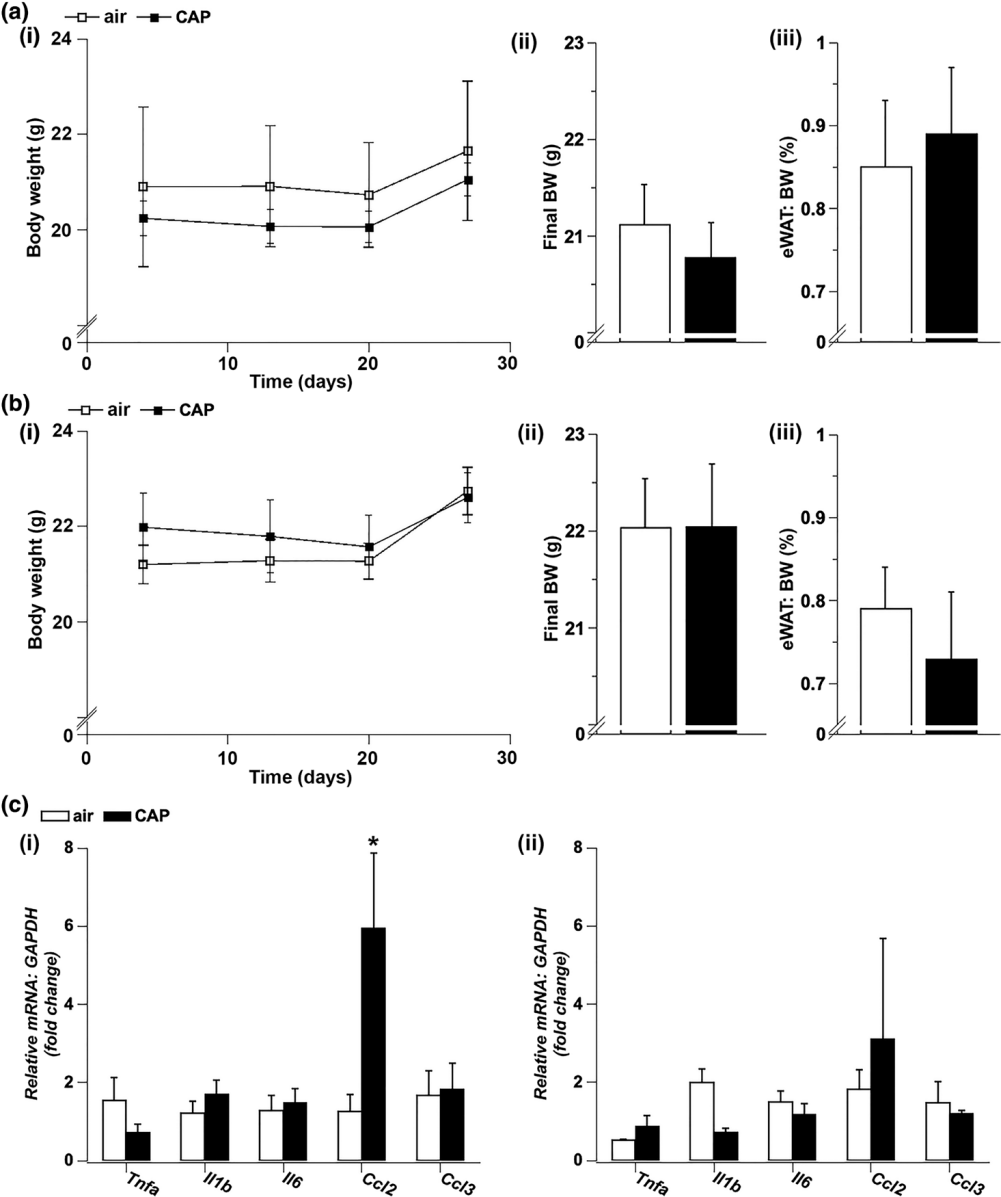
Adiposity and adipose tissue inflammation in female mice. Weekly body weight (BW, i) during the 30 days of exposure, final BW (ii), and epididymal white adipose tissue (eWAT, iii) weight in % of BW measured in female mice maintained on a regular (RLC, a) or switched to a disturbed (DLC, b) light cycle that inhaled air or concentrated ambient fine particulate matter (CAP). Data are presented as mean ± SE, *n* = 10 per group. (c) Abundance of pro‐inflammatory *mRNA* in eWAT of animals kept on an RLC (i) or switched to a DLC (ii) inhaling air or CAP for 30 days. The *mRNA* levels of *Tnfa*, *tumor necrosis factor α*; *Il1b*, *interleukin 1β*; *Il6*, *interleukin 6*; *Ccl2*, *chemokine* (*C‐C motif*) *ligand 2*; *Mcp1*, *monocyte chemoattractant protein‐1*; *Ccl3*, *chemokine* (*C‐C motif*) *ligand 3*; *Mip1α*, *macrophage inflammatory protein 1‐alpha*, were measured by *qRT*‐PCR. Data are normalized to air and presented as mean ± SE, *n* = 5 per group, *n* = 5 per group. Statistical significance differences between air and CAP inhaling mice that were kept either on an RLC or DLC were tested by an unpaired two‐tailed *student's t*‐test and is indicated by * (*p* < 0.05, air vs. CAP).

Next, we examined whether CAP exposure changes body, adiposity or adipose tissue inflammation in female mice kept on a regular (RLC, Figure 3a) or disturbed (DLC, Figure 3b) light cycle. Exposure to CAP neither altered body weights measured during the duration of the experiment (i) nor the body weights measured after the final exposure (ii, Table 1). No changes were observed in the weight of the epididymal white adipose tissue (eWAT) measured in air and CAP inhaling mice kept on an RLC (Figure 3aiii) or DLC (Figure 3biii), indicating that a 30‐day CAP exposure did not induce adiposity. As we have found that CAP exposure increased inflammation in the epididymal white adipose tissue (eWAT) of male mice kept on a disturbed light cycle (Ribble et al., 2023), we examined eWAT inflammation in female mice maintained either on a regular (RLC, Figure 3ci) or disturbed (DLC, Figure 3cii) light cycle that inhaled air or CAP. For this, we measured the abundance of pro‐inflammatory *Tnfα*, *Il1β*, *Il6*, *Ccl2*, and *Ccl3 mRNAs* in the eWAT of female mice kept on an RLC or switched to a DLC that inhaled air or CAP by (*qRT*)*‐PCR*. We found that CAP exposure did not increase the abundance of *Tnfα*, *Il1β*, *Il6*, *Ccl2*, or *Ccl3* in the eWAT of female mice kept on a DLC (Figure 3cii) supporting the idea that female mice on a disturbed light cycle are also protected from CAP‐induced eWAT inflammation.

While CAP exposure had no effect on the abundance of pro‐inflammatory cytokine in the eWAT of female mice kept on a regular light cycle, exposure to CAP increased levels of *Ccl2* (aka *Mcp1*) in the eWAT of female mice kept on an RLC by almost fivefold (RLC‐air: 1.00 ± 0.35 fold, RLC‐CAP: 4.75 ± 1.52 fold, *p* = 0.043, Figure 3ci).

### 3.3 Exposure to PM_2.5_ does not induce cardiac insulin resistance in female mice

**FIGURE 4:**
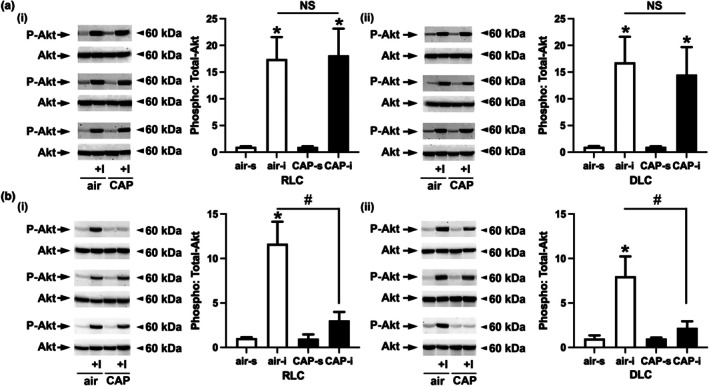
Cardiovascular insulin sensitivity in female and male mice. Western blot analysis of insulin‐stimulated phosphorylation of Akt in the heart of (a) female and (b) male mice kept on a regular light cycle (RLC, i) or placed on a disturbed light cycle (DLC, ii) that inhaled air or concentrated ambient fine particulate matter (CAP) for 30 days. Mice were injected with either saline or insulin (+I, 1.5 U/kg, 15 min). Data are presented as mean ± SE *n* = 5 per group. A two‐way analysis of variance (ANOVA) followed by a Tukey's multiple comparisons test was used to determine statistical significance. Statistical significance between saline and insulin‐injected mice that inhaled either air or CAP is indicated by * (*p* < 0.05, air vs. air‐I, CAP vs. CAP‐I) for each sex (female or male) kept either on an RLC or DLC. Statistically significant differences found between the insulin stimulated phosphorylation of Akt in air or CAP (air‐I vs. CAP‐I) inhaling male mice is indicated by # (*p* < 0.05) for male mice kept either on an RLC or DLC. No differences in the insulin‐stimulated phosphorylation of Akt between air and CAP inhaling female mice kept either on an RLC or DLC are indicated by NS (not significant; RLC: air‐I vs. CAP‐I; DLC: air‐I vs. CAP‐I).

As changes in PM_2.5_‐induced cardiovascular insulin resistance have not yet been examined in female mice, we tested whether a 30‐day CAP exposure impacts cardiac insulin sensitivity. To test whether a 30‐day CAP exposure impairs cardiac insulin signaling in female mice, we measured insulin‐stimulated phosphorylation of Akt in the hearts of female (Figure 4a) and male (Figure 4b) mice kept either on a regular (RLC, i) or disturbed (DLC, ii) light cycle that inhaled air or CAP for 30 days by Western blot. Western blot analysis showed that injection of insulin stimulated the phosphorylation of Akt in the hearts of female mice kept on an RLC (Figure 4ai, air‐I: 17.45 ± 4.11‐fold, CAP‐I: 18.14 ± 5.00‐fold, *p* = 0.998) or DLC (Figure 4aii, air‐I: 16.82 ± 4.80‐fold, CAP‐I: 14.52 ± 5.16‐fold, *p* = 0.953) with no significant differences in phospho‐Akt levels being found between insulin‐injected female mice inhaling air and CAP. In contrast, exposing male mice for 30 days to CAP significantly decreased the insulin‐stimulated phosphorylation of Akt in the hearts of both male mice kept on an RLC (Figure 4bi, air‐I: 11.65 ± 2.47‐fold, CAP‐I: 3.04 ± 0.95‐fold, *p* = 0.003) or DLC (Figure 4bii, air‐I: 8.06 ± 2.22‐fold, CAP‐I: 2.21 ± 0.74‐fold, *p* = 0.012). Taken together, while CAP exposure had no impact on cardiac insulin signaling in female mice kept on an RLC or DLC, CAP exposure impairs insulin signaling in the heart of male mice.

### 3.4 Female mice are protected from PM_2.5_‐induced systemic redox changes

**FIGURE 5:**
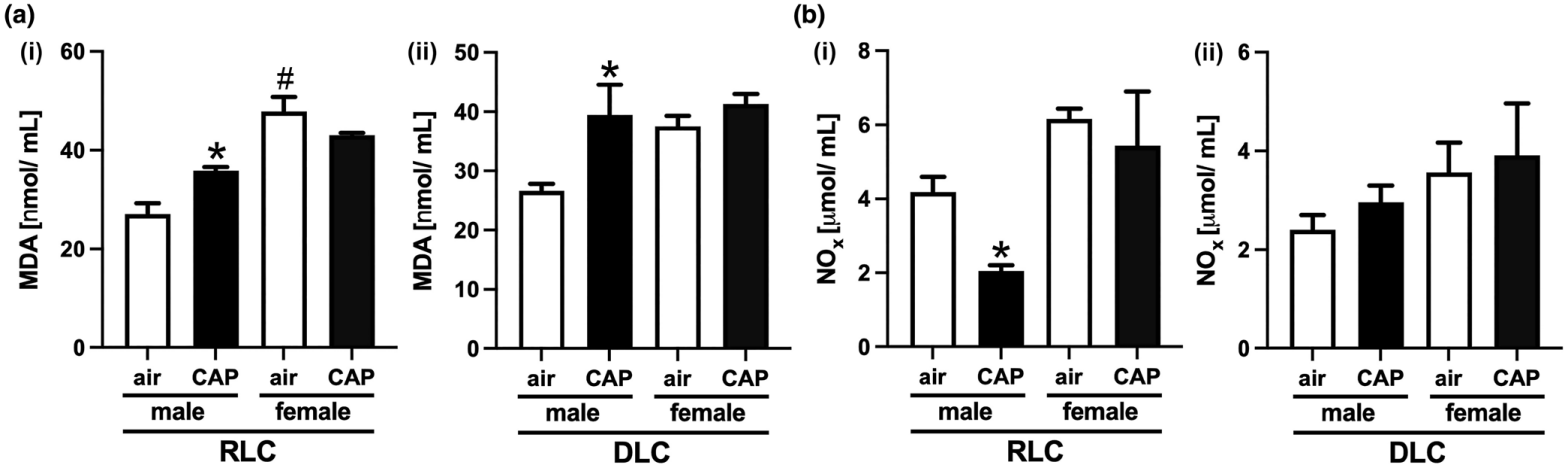
Systemic redox changes in female and male mice. Plasma concentration of (a) malondialdehyde (MDA) and (b) the nitric oxide breakdown product nitrite (NO_x_) in female and male mice kept on a regular light cycle (RLC, i) or placed on a disturbed light cycle (DLC, ii) that inhaled air or concentrated ambient fine particulate matter (CAP) for 30 days. Data are presented as mean ± SE, *n* = 5 per group. A two‐way analysis of variance (ANOVA) followed by a Tukey's multiple comparisons test was used to determine statistical significance. Statistical significance between air and CAP inhaling male mice kept either on an RLC or DLC is indicated by * (*p* < 0.05, air vs. CAP). Statistically significant differences between female and male mice are indicated by # (*p* < 0.05, air‐male vs. air‐female).

Because oxidative and nitrosative stress have been identified to be a major mechanism by which PM_2.5_ impacts cardiovascular and metabolic health (Bhatnagar, 2022; Gangwar et al., 2020; Haberzettl et al., 2014; Singh et al., 2021), we examined CAP‐induced redox changes. To test for systemic redox changes, we measured levels of the lipid peroxidation product malondialdehyde (MDA, Figure 5a) and the abundance of the nitric oxide breakdown product nitrite (NO_x_, Figure 5b) in the plasma of female and male mice maintained on a regular or disturbed light cycle inhaling air or CAP for 30 days. While CAP exposure increased the levels of MDA (air: 27.01 ± 2.27 CAP: 35.82 ± 0.83, *p* = 0.019) and decreased the abundance of NO_x_ (air: 4.18 ± 0.41, CAP: 2.05 ± 0.15, *p* = 0.005) in the plasma of male mice kept on a RLC, CAP exposure did not impact plasma MDA and NO_x_ in female mice, indicating that female mice are protected against PM_2.5_‐induced systemic oxidative/nitrosative stress. However, plasma MDA levels were significantly higher in air inhaling female mice kept on an RLC when compared to their male counterparts (air‐female: 47.87 ± 2.93 vs. air‐male 27.01 ± 2.27, *p* = 0.001). Although not significant, elevated MDA levels are also indicated in female mice kept on RLC that are exposed to CAP (CAP‐female: 43.00 ± 0.45 vs. CAP‐male: 35.82 ± 0.83, *p* = 0.063) and in air‐inhaling female mice switched to a DLC (air‐female: 37.55 ± 1.76 vs. air‐male 26.63 ± 1.16, *p* = 0.063) when compared to their male counterparts. Interestingly, while in male mice kept on a DLC, CAP exposure increased MDA levels (air: 26.63 ± 1.16 CAP: 39.49 ± 5.08, *p* = 0.026), it did not decrease NO_x_ (air: 2.40 ± 0.30, CAP: 2.96 ± 0.34, *p* = 0.912).

### 3.5 Female mice are protected from PM_2.5_‐induced pulmonary redox changes

**FIGURE 6:**
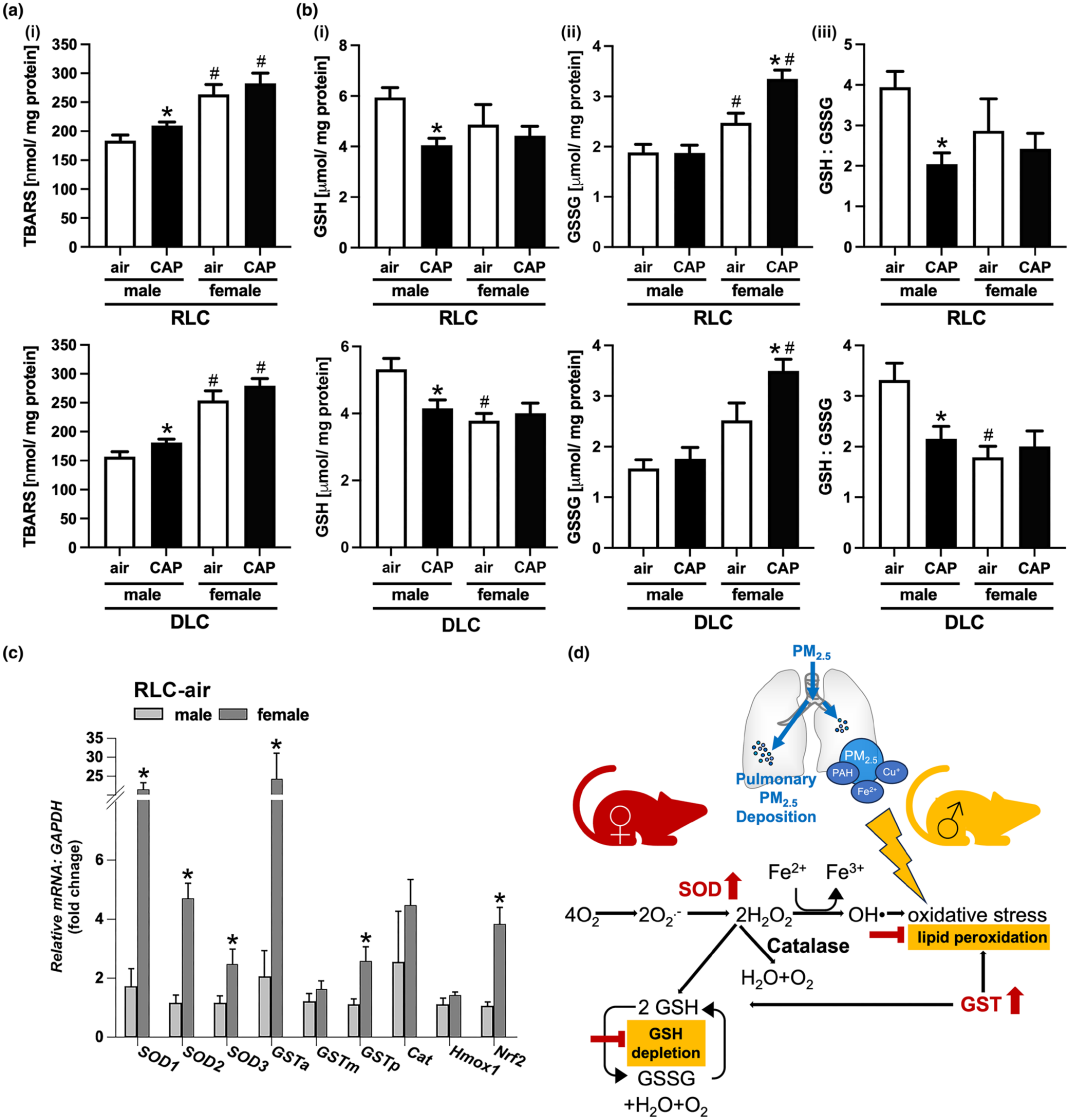
Pulmonary redox changes in female and male mice. Lung levels of (a) thiobarbituric acid reactive substances (TBARS) and (b) reduced glutathione (GSH, i), (oxidized) glutathione disulfide (GSSG, ii), and GSH: GSSG ratios (iii) in female and male mice kept either on a regular light cycle (RLC, upper panel) or placed on a disturbed light cycle (DLC, lower panel) that inhaled air or concentrated ambient fine particulate matter (CAP) for 30 days. Data are presented as mean ± SE, *n* = 5 per group. A two‐way analysis of variance (ANOVA) followed by a Tukey's multiple comparisons test was used to determine statistical significance. Statistical significance between air and CAP inhaling male or female mice kept either on an RLC or DLC is indicated by * (*p* < 0.05, air vs. CAP). Statistically significant differences between female and male mice inhaling air or CAP kept either on an RLC or DLC is indicated by # (*p* < 0.05, air‐male vs. air‐female, CAP male v. CAP‐female). (c) Pulmonary *mRNA* abundance of the antioxidant enzymes *superoxide dismutase* (*Sod1*, *2*, *3*), *glutathione transferase* (*Gst‐a*, *m*, *p*), *catalase* (*Cat*), and *heme oxygenase 1* (*Hmox1*) and their transcription factor *nuclear factor erythroid 2–related factor 2* (*Nrf2*) in air inhaling female and male mice that were kept on an RLC. Data are mean ± SE normalized to male‐ air‐RLC, *n* = 5 per group. Statistical significance between male and female mice was tested by an unpaired two‐tailed *student's t*‐test and is indicated by * (*p* < 0.05, male vs. female). (d) Female mice express more SOD and GST in the lungs and are protected against pulmonary lipid peroxidation and GST depletion induced by the exposure to concentrated PM_2.5_.

Because PM_2.5_‐induced pulmonary oxidative stress has been found to contribute to the development of systemic and tissue‐specific insulin resistance and glucose intolerance (Haberzettl et al., 2018; Haberzettl, O'Toole, et al., 2016; Hill et al., 2021; Kurlawala et al., 2023; Ribble et al., 2023), we examined pulmonary redox differences (Figure 6). First, we assessed CAP‐induced changes in lipid peroxidation in the lungs of female and male mice. For this, we measured the pulmonary abundance of the lipid peroxidation marker thiobarbituric acid reactive substance (TBARS, Figure 6a). Similar to the results obtained from measuring the circulating lipid peroxidation marker MDA (Figure 5a), inhalation of CAP increased the levels of TBARS in the lungs of male mice either kept on an RLC or DLC, whereas CAP exposure did not increase pulmonary lipoperoxidation in female mice. Yet, TBARS levels were elevated in the lungs of female mice across all groups. During oxidative stress in excess produced reactive oxygen species (ROS) are scavenged by glutathione. Reduced glutathione (GSH) is oxidized to glutathione disulfide (GSSG) and the ratio of GSH:GSSG is a commonly used indicator of oxidative stress (Koek et al., 2011). While CAP exposure had no impact on pulmonary GSH levels in female mice regardless of whether the mice were maintained on an RLC or DLC, exposure to CAP decreased GSH levels in the lungs of male mice (Figure 6bi). In contrast, CAP exposure increased the levels of GSSG only in the lungs of female mice (Figure 6bii). These results indicate that female mice are capable of scavenging CAP‐induced pulmonary ROS without depleting GSH that was observed in male mice. This notion is also reflected in the GSH:GSSG ratios that were not impacted in female mice but decreased in the lungs of male mice kept on an RLC or DLC (Figure 6biii). The attenuation of both lipid peroxidation and GSH depletion indicates that female mice are protected against CAP‐induced pulmonary oxidative stress. Such protection would require a higher capacity to scavenge reactive oxygen and nitrogen species by antioxidants, including antioxidant enzymes such as superoxide dismutase (SOD), catalase (Cat), and glutathione S‐transferase (GST) as illustrated in Figure 6d. Consequently, we tested for differences in the *mRNA* abundance of the antioxidant enzymes *Sod1*, *2*, and *3*, *Gsta*, *m*, and *p*, *Cat*, and *Hmox1* and their transcription factor *Nrf2* by *qRT*‐PCR in the lungs of male and female air‐inhaling mice that were kept on an RLC (Figure 6c). Supporting the idea that female mice are less sensitive to PM_2.5_ due to a higher pulmonary antioxidant defense capacity, we found a greater *mRNA* abundance of *Sod1*, *2*, *3*, and *Gsta* and *p* as well as *Nrf2* in female lungs.

## 4 DISCUSSION

The results obtained from the current study will enhance information regarding the health impact of PM_2.5_ exposure in females providing data that are at this point very limited. Our data show that female mice are protected against PM_2.5_‐induced systemic and pulmonary oxidative stress which could aid in the mitigation of the cardiometabolic toxicity of PM_2.5_. Specifically, we found that female mice are protected against the PM_2.5_‐induced exacerbation of glucose intolerance and insulin resistance in SKM and liver in DLC (Figure 2) as previously observed in male mice (Ribble et al., 2023) as well as cardiac insulin resistance (Figure 4), systemic redox changes (Figure 5), and pulmonary oxidative stress (Figure 6) found in both male mice maintained on an RLC or switched to a DLC. Although female mice seem to have a higher redox status than their male counterparts, as indicated by elevated circulating MDA and pulmonary TBARS levels observed across all groups of female mice, female mice were protected against PM_2.5_‐induced pulmonary and systemic oxidative stress. This female protection was accompanied by a higher expression of antioxidant defense enzyme genes in the lungs of female mice when compared to the male counterparts (Figure 6c).

Although our results indicate that female mice are protected against PM_2.5_ by averting pulmonary oxidative stress, other factors could contribute as well. Sex differences in lung size and function could play an important role as such parameters determine the amount of PM_2.5_ that can be inhaled and deposited in the lungs. However, it has been reported that there are no significant differences in lung volumes, breathing frequency, tidal volume, minute ventilation, peak inspiratory flow, and peak expiratory flow between male and female C57/Bl6 mice (Card et al., 2006; Reinhard et al., 2002; Schulz et al., 2002), suggesting that male and female mice inhale similar amounts of PM_2.5_. Taken together, our data suggest that female mice are less sensitive to PM_2.5_‐induced injury than male mice due to a higher pulmonary antioxidant defense capacity that attenuated oxidative stress in the lungs, which is a well‐known contributor to cardiovascular and metabolic toxicity associated with short‐term PM_2.5_ exposure in male mice (Haberzettl et al., 2018; Haberzettl, O'Toole, et al., 2016; Hill et al., 2021; Kurlawala et al., 2023; Ribble et al., 2023).

Further support for our idea that females are protected against PM_2.5_‐induced oxidative stress by a higher capability to cope with generated ROS can be found in the results of human and animal studies that also show increased antioxidant capacity in females as a result of either higher activity or abundance of antioxidant enzymes (Bal et al., 2013; Barp et al., 2002; Bhatia et al., 2012; Borras et al., 2003; Chen et al., 2011; Enomoto et al., 2012; Hanna et al., 2016; Ide et al., 2002; Kim, 2022; Malling et al., 2010; Malorni et al., 2008; Tiberi et al., 2023). For instance, the activity of SOD in the lungs of female mice has been found to be higher than in male mice (Chen et al., 2011). Similar to our findings in mice (Figure 6c), the pulmonary abundance of GST and SOD has been reported to be higher in female than in the male rats (Bal et al., 2013). In addition, GSH showed a higher abundance in the lungs of female rats when compared to male rats (Bal et al., 2013) while it appears that there is no difference in the basal levels of GSH between male and female mice (Figure 6bi). Interestingly, enhanced antioxidant defense in the female lungs was associated with reduced severity of pulmonary hypertension induced by either hypoxia or monocrotaline (Bal et al., 2013; Enomoto et al., 2012) supporting the notion of female protection by an increased antioxidant capacity. In line with these observations, findings of our current study show that female mice with a higher pulmonary *mRNA* abundance of *Sod1*, *2*, *3*, and *Gsta* and *p* are resistant to PM_2.5_‐induced GSH depletion and are protected against the cardiometabolic impact of PM_2.5_.

Taken together, our results show that female mice are protected against the metabolic impact of CAP in mice kept on a disturbed light cycle, such as an increase in systemic glucose intolerance and the induction of SKM and liver insulin resistance that was previously observed in male mice (Ribble et al., 2023). We also found that female mice were protected against eWAT inflammation that was observed in CAP‐exposed male mice kept on a disturbed light cycle (Ribble et al., 2023). Interestingly, while female mice on a DLC were protected against CAP‐induced eWAT inflammation, CAP exposure increased levels of *Ccl2* (aka *Mcp1*) *mRNA* in the eWAT of female mice kept on a RLC (Figure 3ci). As the protein CCL2 is known to contribute to the development of chronic inflammation and the development of the metabolic syndrome (Jiang et al., 2020), an increase in the *Ccl2 mRNA* abundance in the eWAT of CAP‐exposed female mice could reflect an initial step toward the development of insulin resistance observed after longer (24 weeks) exposures to concentrated PM in female mice (Li, Sun, et al., 2020). Although the contribution of adipose tissue inflammation in the development of insulin resistance in female mice after longer CAP exposures needs further investigation, we observed no signs of systemic (Ribble et al., 2023) or tissue‐specific (see Figure 2c,d) insulin resistance in female mice exposed for 30 days to CAP. Interestingly, the observed increase in *Ccl2 mRNA* was still apparent in the eWAT of CAP‐exposed female mice transgenic for the lung‐specific overexpression of extracellular superoxide dismutase (ecSOD‐Tg, data not shown). While preventing CAP‐induced pulmonary oxidative stress in male ecSOD‐Tg mice protected from cardiovascular and metabolic injury (Haberzettl, O'Toole, et al., 2016; Hill et al., 2021; Kurlawala et al., 2023; Ribble et al., 2023), it did not avert the increase in *Ccl2‐mRNA* abundance in the adipose tissue of CAP‐exposed female mice. This indicates that the CAP‐induced increase in *Ccl2‐mRNA* in the eWAT of female mice seems to be induced by a mechanism independent of pulmonary oxidative stress.

The current study indicates that female mice are protected against PM_2.5_‐cardiotoxicity. In contrast, growing epidemiological evidence indicates that the CVD risk associated with air pollution exposure is higher in women than in men. As such, exposure to elevated levels of ambient PM_2.5_ increases hospital admission for CVDs at a higher rate in women than in men (Bell et al., 2015; Su et al., 2016) and associations between PM_2.5_ and cardiovascular mortality, hypertension, and atherosclerosis are stronger in the female population (Adar et al., 2013; Cheng et al., 2019; Curto et al., 2019). Similar associations have been found between the exposure to PM_2.5_ and the risk to develop T2D. Foor instnace, a population study indicates that the impact of PM_2.5_ exposure on the risk to develop T2D is stronger in females (Mazidi & Speakman, 2017). While associations between PM_2.5_ and hypertension were found to be stronger in females (Curto et al., 2019), the risk for hypertension associated with PM_2.5_ exposure is lower in women at reproductive age (18–45 years of age), an age at which men are at a higher risk (Wang et al., 2020; Xie et al., 2018). This shows that age is an important factor to be considered when investigating sex‐specific differences in the sensitivity to PM_2.5_. Like in our animal study with young female mice, only younger women seem to be protected against PM_2.5_ indicating that age‐related changes such as the onset of menopause could impact PM_2.5_ sensitivity. This is supported by the observation that the atherosclerosis risk associated with air pollution exposure is higher in postmenopausal women (Wang et al., 2019). The finding that young women are at a lower risk than men or postmenopausal women indicates a possible protective role of estrogen. Interestingly, estrogen has been found to increase the levels of GSH and antioxidant enzymes (i.e., SOD and HO‐1) and to induce nuclear factor erythroid 2–related factor 2 (Nrf2) signaling that regulates the transcription of several antioxidants (Borras et al., 2003; Hanna et al., 2016; Malorni et al., 2008). Hence, estrogen by increasing levels of GSH and SOD could play an important role in the female protection against PM_2.5_. Although future studies still have to determine whether and how estrogen contributes to the advanced pulmonary antioxidant defense and reduces the PM_2.5_ sensitivity, an animal study showing that ovariectomy decreased the antioxidant defense against PM_2.5_ (Goettems‐Fiorin et al., 2019) supports the idea that estrogen enhances the pulmonary protection against PM_2.5_. Ovariectomy and a decline in the pulmonary antioxidant defense were associated with an increase in pro‐inflammatory responses in the lungs (Goettems‐Fiorin et al., 2019). Additionally, other studies have shown that ovariectomy also impacts the cardiometabolic responses to residual fly ash (ROFA) a combustion byproduct that contributes to the particulate matter in the ambient air (Costa‐Beber, Goettems‐Fiorin, Dos Santos, Friske, Frizzo, et al., 2021; Costa‐Beber, Goettems‐Fiorin, Dos Santos, Friske, Heck, et al., 2021). The lack of estrogen after ovariectomy, which was found to be associated with an impaired antioxidant defense response to particulate matter air pollution, could by increasing oxidative stress in the lungs contribute to pulmonary, cardiovascular, and metabolic injury. Interestingly, changes in estrogen have also been associated with the incidence and progression of cardiovascular disease (CVD) and type 2 diabetes (T2D) independent of air pollution exposures. While diabetic cardiovascular complications are more severe in women, sex‐specific differences seem to put men at a higher risk to develop CVD and T2D than women. As such, sex differences result in an estimate of 18 million more T2D cases in men than in women (Ciarambino et al., 2022; Kautzky‐Willer et al., 2023). However, the difference between women and men to develop T2D becomes more similar during aging when estrogen levels decrease in women. Because estrogen has been reported to contribute to the regulation of insulin signaling, a decrease in estrogen levels during menopause could be a major factor that increases the T2D risk. This is supported by the finding that estrogen treatment during menopause improves insulin sensitivity and consequently glucose handling (Paschou et al., 2019). Although further experiments are required, the presence of estrogen could by improving insulin sensitivity protect female mice from the impact of PM_2.5_ on glucose homeostasis independent of preventing pulmonary oxidative stress. However, the process seems to be more complex as a recent study on the risk for T2D in an aged population (~63 years) including postmenopausal women shows stronger association between PM_2.5_ exposure and T2D in men than in women (Niedermayer et al., 2024).

While female mice kept on a DLC were protected from the impact of CAP on glucose homeostasis, female sex did not protect against glucose intolerance that resulted from a disturbed light cycle (Figure 2b). Extending the light cycle increased the calculated area under the curve (AUC) during GTT in both female mice that inhaled either air or CAP (Figure 2b). In contrast, other studies indicate that female mice seem to be protected against glucose intolerance and insulin resistance in other rodent models of circadian disturbances such as jet lag (Zhu et al., 2015), shift work (Zhong et al., 2019), or exposure to an extended dark cycle (Tan et al., 2024). The protection against jet lag seems to be at least in part mediated by estrogens, as estrogen treatment prevented glucose intolerance and insulin resistance in ovariectomized mice subjected to the jet lag protocol (Zhu et al., 2015). Similarly, female sex hormones have been demonstrated to protect against the effects of phase shift induced circadian disruption (Het Panhuis et al., 2023). Regardless, data regarding the sex‐specific development of glucose intolerance and insulin resistance due to circadian disturbances are still limited, and further research is required to determine the role of sex hormones in the development of the metabolic syndrome and T2D related to circadian disturbances.

Limitations of this study are that (1) the role of estrogens in the female protection against air pollution exposure is not assessed, (2) the impact of aging is not evaluated, (3) effects of long‐term exposure are not investigated, and (4) other susceptibility states (i.e., diet‐induced obesity) are not examined. A major limit of the current study is that a thorough assessment of whether estrogen and estrogen signaling contributes to the female protection is lacking. Measuring estrogen and estrogen signaling in exposed mice, performing inhalation studies that utilize ovariectomized mice and mice of different ages as well as executing experiments that test for the effects of estrogen treatments in exposed mice would provide further evidence for the notion that estrogen plays a crucial role in the defense against PM_2.5_. Although important to untangle the time course and consequently the mechanism, we only tested for the effects of relatively short exposure durations. In contrast to our short‐term exposure study, long‐term PM_2.5_ exposure has been found to impair systemic insulin sensitivity in male and female mice (Li, Sun, et al., 2020). While the 24‐week exposure to concentrated PM_2.5_ impairs insulin sensitivity, 30‐day exposure neither induced glucose intolerance nor insulin resistance in male or female mice. Short‐term exposure induced cardiac insulin resistance in male mice but not in female mice. Moreover, while in male mice either fed a high fat diet or maintained on a disturbed light cycle, CAP exacerbated glucose intolerance and insulin resistance (Haberzettl, O'Toole, et al., 2016; Ribble et al., 2023), female mice kept on a DLC were protected against the effects of short‐term PM_2.5_ exposure on glucose homeostasis. Additional studies are needed that test whether and how the female protection faints during long‐term exposure and to investigate whether diet‐induced obesity increases the susceptibility to PM_2.5_ in female mice.

## 5 CONCLUSION

Taken together, we found that female mice were protected against the impact of PM_2.5_ on glucose tolerance in DLC as well as PM_2.5_‐induced cardiac insulin resistance, systemic oxidative/nitrosative stress, and pulmonary redox changes. This female protection was accompanied by a higher expression of antioxidant enzymes in the female lungs indicating that the female protection is a result of a better pulmonary antioxidant defense mechanism (see Figure 6d). In addition, the findings of the current experiments in female mice combined with the results from our previous studies in male mice (Haberzettl, O'Toole, et al., 2016; Ribble et al., 2023) support the notion that PM_2.5_, by inducing pulmonary oxidative stress, promotes cardiometabolic injury. Regardless, further research is required to identify sex‐specific differences in the health impact of short‐ and long‐term PM_2.5_ exposures. Moreover, other factors such as unhealthy lifestyle choices (e.g., diet, sleep habits) or age that differentially impact the health effects of air pollution in females and males need to be recognized. Such studies would not only help to provide sex‐specific recommendations and prevention strategies but also help to identify the mechanism(s) by which exposure to polluted air impacts cardiometabolic health.

## AUTHOR CONTRIBUTIONS

The manuscript was written through contributions of all authors. All authors have given approval to the final version of the manuscript. M.K. performed experiments, analyzed data, prepared figures, drafted the manuscript, prepared figures. A.R. performed experiments, analyzed data, prepared figures, drafted the manuscript. A.W. performed experiments, analyzed data, prepared figures, drafted the manuscript. A.B. edited and revised the manuscript, conceptualization. P.H. conceived and designed research, performed experiments, analyzed data, interpreted results of experiments, prepared figures, edited and revised the manuscript.

## FUNDING INFORMATION

This work was supported by grants from the National Institute of Health: ES027881, ES028268, GM127607, T35ES014559.

## CONFLICT OF INTEREST STATEMENT

The authors declare that they have no known competing financial interests or personal relationships that could have appeared to influence the work reported in this paper.

## Data Availability

Data will be made available upon reasonable request.

## References

[phy270536-bib-0001] Adar, S. D. , Sheppard, L. , Vedal, S. , Polak, J. F. , Sampson, P. D. , Diez Roux, A. V. , Budoff, M. , Jacobs, D. R., Jr. , Barr, R. G. , Watson, K. , & Kaufman, J. D. (2013). Fine particulate air pollution and the progression of carotid intima‐medial thickness: A prospective cohort study from the multi‐ethnic study of atherosclerosis and air pollution. PLoS Medicine, 10, e1001430. 23637576 10.1371/journal.pmed.1001430 PMC3637008

[phy270536-bib-0021] Aguilar Diaz De Leon, J. , & Borges, C. R. (2020). Evaluation of oxidative stress in biological samples using the thiobarbituric acid reactive substances assay. Journal of Visualized Experiments, (159). 10.3791/61122 PMC9617585 32478759

[phy270536-bib-0002] Bal, E. , Ilgin, S. , Atli, O. , Ergun, B. , & Sirmagul, B. (2013). The effects of gender difference on monocrotaline‐induced pulmonary hypertension in rats. Human & Experimental Toxicology, 32(7), 766–774. 23821593 10.1177/0960327113477874

[phy270536-bib-0003] Barp, J. , Araujo, A. S. , Fernandes, T. R. , Rigatto, K. V. , Llesuy, S. , Bello‐Klein, A. , & Singal, P. (2002). Myocardial antioxidant and oxidative stress changes due to sex hormones. Brazilian Journal of Medical and Biological Research, 35, 1075–1081. 12219179 10.1590/s0100-879x2002000900008

[phy270536-bib-0004] Bell, M. L. , Son, J. Y. , Peng, R. D. , Wang, Y. , & Dominici, F. (2015). Ambient PM2.5 and risk of hospital admissions: Do risks differ for men and women? Epidemiology, 26, 575–579. 25906368 10.1097/EDE.0000000000000310 PMC4452416

[phy270536-bib-0005] Bhatia, K. , Elmarakby, A. A. , El‐Remessy, A. B. , & Sullivan, J. C. (2012). Oxidative stress contributes to sex differences in angiotensin II‐mediated hypertension in spontaneously hypertensive rats. American Journal of Physiology. Regulatory, Integrative and Comparative Physiology, 302, R274–R282. 22049231 10.1152/ajpregu.00546.2011 PMC3349386

[phy270536-bib-0006] Bhatnagar, A. (2022). Cardiovascular effects of particulate air pollution. Annual Review of Medicine, 73, 393–406. 10.1146/annurev-med-042220-011549 PMC10132287 34644154

[phy270536-bib-0007] Borras, C. , Sastre, J. , Garcia‐Sala, D. , Lloret, A. , Pallardo, F. V. , & Vina, J. (2003). Mitochondria from females exhibit higher antioxidant gene expression and lower oxidative damage than males. Free Radical Biology & Medicine, 34, 546–552. 12614843 10.1016/s0891-5849(02)01356-4

[phy270536-bib-0008] Brook, R. D. , Xu, X. , Bard, R. L. , Dvonch, J. T. , Morishita, M. , Kaciroti, N. , Sun, Q. , Harkema, J. , & Rajagopalan, S. (2013). Reduced metabolic insulin sensitivity following sub‐acute exposures to low levels of ambient fine particulate matter air pollution. The Science of the Total Environment, 448, 66–71. 22901427 10.1016/j.scitotenv.2012.07.034 PMC4391076

[phy270536-bib-0009] Burke, M. , Childs, M. L. , de la Cuesta, B. , Qiu, M. , Li, J. , Gould, C. F. , Heft‐Neal, S. , & Wara, M. (2023). The contribution of wildfire to PM(2.5) trends in the USA. Nature, 622, 761–766. 37730996 10.1038/s41586-023-06522-6

[phy270536-bib-0010] Card, J. W. , Carey, M. A. , Bradbury, J. A. , DeGraff, L. M. , Morgan, D. L. , Moorman, M. P. , Flake, G. P. , & Zeldin, D. C. (2006). Gender differences in murine airway responsiveness and lipopolysaccharide‐induced inflammation. Journal of Immunology, 177, 621–630. 10.4049/jimmunol.177.1.621 PMC2262913 16785560

[phy270536-bib-0011] Chen, Y. , Ji, L. L. , Liu, T. Y. , & Wang, Z. T. (2011). Evaluation of gender‐related differences in various oxidative stress enzymes in mice. The Chinese Journal of Physiology, 54, 385–390. 22229505 10.4077/CJP.2011.AMM080

[phy270536-bib-0012] Cheng, H. , Zhu, F. , Lei, R. , Shen, C. , Liu, J. , Yang, M. , Ding, R. , & Cao, J. (2019). Associations of ambient PM(2.5) and O(3) with cardiovascular mortality: A time‐series study in Hefei, China. International Journal of Biometeorology, 63, 1437–1447. 31385092 10.1007/s00484-019-01766-2

[phy270536-bib-0013] Ciarambino, T. , Crispino, P. , Leto, G. , Mastrolorenzo, E. , Para, O. , & Giordano, M. (2022). Influence of gender in diabetes mellitus and its complication. International Journal of Molecular Sciences, 23, 8850. 36012115 10.3390/ijms23168850 PMC9408508

[phy270536-bib-0014] Cipak Gasparovic, A. , Zarkovic, N. , Zarkovic, K. , Semen, K. , Kaminskyy, D. , Yelisyeyeva, O. , & Bottari, S. P. (2017). Biomarkers of oxidative and nitro‐oxidative stress: Conventional and novel approaches. British Journal of Pharmacology, 174, 1771–1783. 27864827 10.1111/bph.13673 PMC5446576

[phy270536-bib-0015] Coogan, P. F. , White, L. F. , Jerrett, M. , Brook, R. D. , Su, J. G. , Seto, E. , Burnett, R. , Palmer, J. R. , & Rosenberg, L. (2012). Air pollution and incidence of hypertension and diabetes mellitus in black women living in Los Angeles. Circulation, 125, 767–772. 22219348 10.1161/CIRCULATIONAHA.111.052753 PMC3326581

[phy270536-bib-0016] Cordiano, R. , Di Gioacchino, M. , Mangifesta, R. , Panzera, C. , Gangemi, S. , & Minciullo, P. L. (2023). Malondialdehyde as a potential oxidative stress marker for allergy‐oriented diseases: An update. Molecules, 28, 5979. 37630231 10.3390/molecules28165979 PMC10457993

[phy270536-bib-0017] Costa‐Beber, L. C. , Goettems‐Fiorin, P. B. , Dos Santos, J. B. , Friske, P. T. , Frizzo, M. N. , Heck, T. G. , Hirsch, G. E. , & Ludwig, M. S. (2021). Ovariectomy enhances female rats' susceptibility to metabolic, oxidative, and heat shock response effects induced by a high‐fat diet and fine particulate matter. Experimental Gerontology, 145, 111215. 33340683 10.1016/j.exger.2020.111215

[phy270536-bib-0018] Costa‐Beber, L. C. , Goettems‐Fiorin, P. B. , Dos Santos, J. B. , Friske, P. T. , Heck, T. G. , Hirsch, G. E. , & Ludwig, M. S. (2021). Ovariectomy reduces the cardiac cytoprotection in rats exposed to particulate air pollutant. Environmental Science and Pollution Research International, 28, 23395–23404. 33443732 10.1007/s11356-021-12350-w

[phy270536-bib-0019] Curto, A. , Wellenius, G. A. , Mila, C. , Sanchez, M. , Ranzani, O. , Marshall, J. D. , Kulkarni, B. , Bhogadi, S. , Kinra, S. , & Tonne, C. (2019). Ambient particulate air pollution and blood pressure in peri‐urban India. Epidemiology, 30, 492–500. 31162282 10.1097/EDE.0000000000001014 PMC6558270

[phy270536-bib-0020] da Silva Rosa, S. C. , Nayak, N. , Caymo, A. M. , & Gordon, J. W. (2020). Mechanisms of muscle insulin resistance and the cross‐talk with liver and adipose tissue. Physiological Reports, 8, e14607. 33038072 10.14814/phy2.14607 PMC7547588

[phy270536-bib-0022] Enomoto, M. , Gosal, K. , Cubells, E. , Escobar, J. , Vento, M. , Jankov, R. P. , & Belik, J. (2012). Sex‐dependent changes in the pulmonary vasoconstriction potential of newborn rats following short‐term oxygen exposure. Pediatric Research, 72, 468–478. 22926548 10.1038/pr.2012.120

[phy270536-bib-0023] Fuller, R. , Landrigan, P. J. , Balakrishnan, K. , Bathan, G. , Bose‐O'Reilly, S. , Brauer, M. , Caravanos, J. , Chiles, T. , Cohen, A. , Corra, L. , Cropper, M. , Ferraro, G. , Hanna, J. , Hanrahan, D. , Hu, H. , Hunter, D. , Janata, G. , Kupka, R. , Lanphear, B. , … Yan, C. (2022). Pollution and health: A progress update. Lancet Planetary Health, 6, e535–e547. 35594895 10.1016/S2542-5196(22)00090-0 PMC11995256

[phy270536-bib-0024] Gangwar, R. S. , Bevan, G. H. , Palanivel, R. , Das, L. , & Rajagopalan, S. (2020). Oxidative stress pathways of air pollution mediated toxicity: Recent insights. Redox Biology, 34, 101545. 32505541 10.1016/j.redox.2020.101545 PMC7327965

[phy270536-bib-0025] Goettems‐Fiorin, P. B. , Costa‐Beber, L. C. , Dos Santos, J. B. , Friske, P. T. , Sulzbacher, L. M. , Frizzo, M. N. , Ludwig, M. S. , Rhoden, C. R. , & Heck, T. G. (2019). Ovariectomy predisposes female rats to fine particulate matter exposure's effects by altering metabolic, oxidative, pro‐inflammatory, and heat‐shock protein levels. Environmental Science and Pollution Research International, 26, 20581–20594. 31104233 10.1007/s11356-019-05383-9

[phy270536-bib-0026] Haberzettl, P. , Bhatnagar, A. , & Conklin, D. J. (2014). Particulate matter and oxidative stress–pulmonary and cardiovascular targets and consequences. In Systems Biology of Free Radicals and Antioxidants (pp. 1557–1586). Springer Berlin Heidelberg.

[phy270536-bib-0027] Haberzettl, P. , Conklin, D. J. , Abplanalp, W. T. , Bhatnagar, A. , & O'Toole, T. E. (2018). Inhalation of fine particulate matter impairs endothelial progenitor cell function via pulmonary oxidative stress. Arteriosclerosis, Thrombosis, and Vascular Biology, 38, 131–142. 29191925 10.1161/ATVBAHA.117.309971 PMC5746456

[phy270536-bib-0028] Haberzettl, P. , McCracken, J. P. , Bhatnagar, A. , & Conklin, D. J. (2016). Insulin sensitizers prevent fine particulate matter‐induced vascular insulin resistance and changes in endothelial progenitor cell homeostasis. American Journal of Physiology. Heart and Circulatory Physiology, 310, H1423–H1438. 27016579 10.1152/ajpheart.00369.2015 PMC4971897

[phy270536-bib-0029] Haberzettl, P. , O'Toole, T. E. , Bhatnagar, A. , & Conklin, D. J. (2016). Exposure to fine particulate air pollution causes vascular insulin resistance by inducing pulmonary oxidative stress. Environmental Health Perspectives, 124, 1830–1839. 27128347 10.1289/EHP212 PMC5132639

[phy270536-bib-0030] Hanna, D. , Riedmaier, A. E. , Sugamori, K. S. , & Grant, D. M. (2016). Influence of sex and developmental stage on acute hepatotoxic and inflammatory responses to liver procarcinogens in the mouse. Toxicology, 373, 30–40. 27746196 10.1016/j.tox.2016.10.006

[phy270536-bib-0031] Hatting, M. , Tavares, C. D. J. , Sharabi, K. , Rines, A. K. , & Puigserver, P. (2018). Insulin regulation of gluconeogenesis. Annals of the New York Academy of Sciences, 1411, 21–35. 28868790 10.1111/nyas.13435 PMC5927596

[phy270536-bib-0032] Het Panhuis, W. , Schonke, M. , Siebeler, R. , Banen, D. , Pronk, A. C. M. , Streefland, T. C. M. , Afkir, S. , Sips, H. C. M. , Kroon, J. , Rensen, P. C. N. , & Kooijman, S. (2023). Circadian disruption impairs glucose homeostasis in male but not in female mice and is dependent on gonadal sex hormones. FASEB Journal: Official Publication of the Federation of American Societies for Experimental Biology, 37, e22772. 36645117 10.1096/fj.202201586R

[phy270536-bib-0033] Hill, B. G. , Rood, B. , Ribble, A. , & Haberzettl, P. (2021). Fine particulate matter (PM2.5) inhalation‐induced alterations in the plasma lipidome as promoters of vascular inflammation and insulin resistance. American Journal of Physiology Heart and Circulatory Physiology, 320, H1836–H1850. 33666505 10.1152/ajpheart.00881.2020 PMC8163652

[phy270536-bib-0034] Hu, X. , Nie, Z. , Ou, Y. , Lin, L. , Qian, Z. , Vaughn, M. G. , McMillin, S. E. , Zhou, Y. , Wu, Y. , Dong, G. , & Dong, H. (2023). Long‐term exposure to ambient air pollution, circadian syndrome and cardiovascular disease: A nationwide study in China. The Science of the Total Environment, 868, 161696. 36682545 10.1016/j.scitotenv.2023.161696

[phy270536-bib-0035] Humphrey, J. L. , Kinnee, E. J. , Robinson, L. F. , & Clougherty, J. E. (2024). Disentangling impacts of multiple pollutants on acute cardiovascular events in New York city: A case‐crossover analysis. Environmental Research, 242, 117758. 38029813 10.1016/j.envres.2023.117758 PMC11378578

[phy270536-bib-0036] Ide, T. , Tsutsui, H. , Ohashi, N. , Hayashidani, S. , Suematsu, N. , Tsuchihashi, M. , Tamai, H. , & Takeshita, A. (2002). Greater oxidative stress in healthy young men compared with premenopausal women. Arteriosclerosis, Thrombosis, and Vascular Biology, 22, 438–442. 11884287 10.1161/hq0302.104515

[phy270536-bib-0037] Jiang, Q. , Maresch, C. C. , Petry, S. F. , Paradowska‐Dogan, A. , Bhushan, S. , Chang, Y. , Wrenzycki, C. , Schuppe, H. C. , Houska, P. , Hartmann, M. F. , Wudy, S. A. , Shi, L. , & Linn, T. (2020). Elevated CCL2 causes Leydig cell malfunction in metabolic syndrome. JCI Insight, 5(21), e134882. 33148888 10.1172/jci.insight.134882 PMC7710294

[phy270536-bib-0038] Jones, D. P. (2002). Redox potential of GSH/GSSG couple: Assay and biological significance. Methods in Enzymology, 348, 93–112. 11885298 10.1016/s0076-6879(02)48630-2

[phy270536-bib-0039] Kautzky‐Willer, A. , Leutner, M. , & Harreiter, J. (2023). Sex differences in type 2 diabetes. Diabetologia, 66, 986–1002. 36897358 10.1007/s00125-023-05891-x PMC10163139

[phy270536-bib-0040] Kim, S. Y. (2022). Oxidative stress and gender disparity in cancer. Free Radical Research, 56, 90–105. 35118928 10.1080/10715762.2022.2038789

[phy270536-bib-0041] Klompmaker, J. O. , Hart, J. E. , Dominici, F. , James, P. , Roscoe, C. , Schwartz, J. , Yanosky, J. D. , Zanobetti, A. , & Laden, F. (2024). Associations of fine particulate matter with incident cardiovascular disease; comparing models using ZIP code‐level and individual‐level fine particulate matter and confounders. The Science of the Total Environment, 926, 171866. 38521279 10.1016/j.scitotenv.2024.171866 PMC11034806

[phy270536-bib-0042] Koek, G. H. , Liedorp, P. R. , & Bast, A. (2011). The role of oxidative stress in non‐alcoholic steatohepatitis. Clinica Chimica Acta; International Journal of Clinical Chemistry, 412, 1297–1305. 21514287 10.1016/j.cca.2011.04.013

[phy270536-bib-0043] Kramer, U. , Herder, C. , Sugiri, D. , Strassburger, K. , Schikowski, T. , Ranft, U. , & Rathmann, W. (2010). Traffic‐related air pollution and incident type 2 diabetes: Results from the SALIA cohort study. Environmental Health Perspectives, 118, 1273–1279. 20504758 10.1289/ehp.0901689 PMC2944089

[phy270536-bib-0044] Kubota, T. , Kubota, N. , Kumagai, H. , Yamaguchi, S. , Kozono, H. , Takahashi, T. , Inoue, M. , Itoh, S. , Takamoto, I. , Sasako, T. , Kumagai, K. , Kawai, T. , Hashimoto, S. , Kobayashi, T. , Sato, M. , Tokuyama, K. , Nishimura, S. , Tsunoda, M. , Ide, T. , … Kadowaki, T. (2011). Impaired insulin signaling in endothelial cells reduces insulin‐induced glucose uptake by skeletal muscle. Cell Metabolism, 13, 294–307. 21356519 10.1016/j.cmet.2011.01.018

[phy270536-bib-0045] Kurlawala, Z. , Singh, P. , Hill, B. G. , & Haberzettl, P. (2023). Fine particulate matter (PM2.5)‐induced pulmonary oxidative stress contributes to changes in the plasma lipidome and liver transcriptome in mice. Toxicological sciences: an official journal of the Society of Toxicology, 192, 209–222. 36857595 10.1093/toxsci/kfad020 PMC10109534

[phy270536-bib-0046] Li, R. , Sun, Q. , Lam, S. M. , Chen, R. , Zhu, J. , Gu, W. , Zhang, L. , Tian, H. , Zhang, K. , Chen, L. C. , Sun, Q. , Shui, G. , & Liu, C. (2020). Sex‐dependent effects of ambient PM(2.5) pollution on insulin sensitivity and hepatic lipid metabolism in mice. Particle and Fibre Toxicology, 17, 14. 32321544 10.1186/s12989-020-00343-5 PMC7178763

[phy270536-bib-0047] Li, R. , Wang, Y. , Chen, R. , Gu, W. , Zhang, L. , Gu, J. , Wang, Z. , Liu, Y. , Sun, Q. , Zhang, K. , & Liu, C. (2020). Ambient fine particulate matter disrupts hepatic circadian oscillation and lipid metabolism in a mouse model. Environmental Pollution, 262, 114179. 32145476 10.1016/j.envpol.2020.114179

[phy270536-bib-0048] Li, X. , Wang, M. , Song, Y. , Ma, H. , Zhou, T. , Liang, Z. , & Qi, L. (2021). Obesity and the relation between joint exposure to ambient air pollutants and incident type 2 diabetes: A cohort study in UK biobank. PLoS Medicine, 18, e1003767. 34460827 10.1371/journal.pmed.1003767 PMC8439461

[phy270536-bib-0049] Liao, M. , Braunstein, Z. , & Rao, X. (2023). Sex differences in particulate air pollution‐related cardiovascular diseases: A review of human and animal evidence. The Science of the Total Environment, 884, 163803. 37137360 10.1016/j.scitotenv.2023.163803

[phy270536-bib-0050] Liu, W. , Song, J. , Yu, L. , Lai, X. , Shi, D. , Fan, L. , Wang, H. , Yang, Y. , Liang, R. , Wan, S. , Zhang, Y. , & Wang, B. (2024). Exposure to ambient air pollutants during circadian syndrome and subsequent cardiovascular disease and its subtypes and death: A trajectory analysis. The Science of the Total Environment, 944, 173777. 38844213 10.1016/j.scitotenv.2024.173777

[phy270536-bib-0051] Liu, X. , Xiao, Y. , Zhu, Q. , Cui, Y. , Hao, H. , Wang, M. , Cowan, P. J. , Korthuis, R. J. , Li, G. , Sun, Q. , & Liu, Z. (2021). Circulating endothelial progenitor cells are preserved in female mice exposed to ambient fine particulate matter independent of estrogen. International Journal of Molecular Sciences, 22, 7200. 34281260 10.3390/ijms22137200 PMC8268796

[phy270536-bib-0052] Malling, T. H. , Sigsgaard, T. , Andersen, H. R. , Deguchi, Y. , Brandslund, I. , Skadhauge, L. , Thomsen, G. , Baelum, J. , Sherson, D. , & Omland, O. (2010). Differences in associations between markers of antioxidative defense and asthma are sex specific. Gender Medicine, 7, 115–124. 20435274 10.1016/j.genm.2010.03.004

[phy270536-bib-0053] Malorni, W. , Straface, E. , Matarrese, P. , Ascione, B. , Coinu, R. , Canu, S. , Galluzzo, P. , Marino, M. , & Franconi, F. (2008). Redox state and gender differences in vascular smooth muscle cells. FEBS Letters, 582, 635–642. 18242172 10.1016/j.febslet.2008.01.034

[phy270536-bib-0054] Mazidi, M. , & Speakman, J. R. (2017). Ambient particulate air pollution (PM2.5) is associated with the ratio of type 2 diabetes to obesity. Scientific Reports, 7, 9144. 28831041 10.1038/s41598-017-08287-1 PMC5567252

[phy270536-bib-0055] Niedermayer, F. , Wolf, K. , Zhang, S. , Dallavalle, M. , Nikolaou, N. , Schwettmann, L. , Selsam, P. , Hoffmann, B. , Schneider, A. , & Peters, A. (2024). Sex‐specific associations of environmental exposures with prevalent diabetes and obesity—results from the KORA fit study. Environmental Research, 252, 118965. 38642640 10.1016/j.envres.2024.118965

[phy270536-bib-0056] Palanivel, R. , Vinayachandran, V. , Biswal, S. , Deiuliis, J. A. , Padmanabhan, R. , Park, B. , Gangwar, R. S. , Durieux, J. C. , Ebreo Cara, E. A. , Das, L. , Bevan, G. , Fayad, Z. A. , Tawakol, A. , Jain, M. K. , Rao, S. , & Rajagopalan, S. (2020). Exposure to air pollution disrupts circadian rhythm through alterations in chromatin Dynamics. iScience, 23, 101728. 33241196 10.1016/j.isci.2020.101728 PMC7672280

[phy270536-bib-0057] Paschou, S. A. , Anagnostis, P. , Pavlou, D. I. , Vryonidou, A. , Goulis, D. G. , & Lambrinoudaki, I. (2019). Diabetes in Menopause: Risks and Management. Current Vascular Pharmacology, 17, 556–563. 29938620 10.2174/1570161116666180625124405

[phy270536-bib-0058] Pearson, J. F. , Bachireddy, C. , Shyamprasad, S. , Goldfine, A. B. , & Brownstein, J. S. (2010). Association between fine particulate matter and diabetes prevalence in the U.S. Diabetes Care, 33, 2196–2201. 20628090 10.2337/dc10-0698 PMC2945160

[phy270536-bib-0059] Qin, G. , Xia, J. , Zhang, Y. , Guo, L. , Chen, R. , & Sang, N. (2018). Ambient fine particulate matter exposure induces reversible cardiac dysfunction and fibrosis in juvenile and older female mice. Particle and Fibre Toxicology, 15, 27. 29941001 10.1186/s12989-018-0264-2 PMC6019275

[phy270536-bib-0060] Rajagopalan, S. , Brook, R. D. , Salerno, P. , Bourges‐Sevenier, B. , Landrigan, P. , Nieuwenhuijsen, M. J. , Munzel, T. , Deo, S. V. , & Al‐Kindi, S. (2024). Air pollution exposure and cardiometabolic risk. The Lancet Diabetes and Endocrinology, 12, 196–208. 38310921 10.1016/S2213-8587(23)00361-3 PMC11264310

[phy270536-bib-0061] Rajagopalan, S. , & Landrigan, P. J. (2021). Pollution and the heart. The New England Journal of Medicine, 385, 1881–1892. 34758254 10.1056/NEJMra2030281

[phy270536-bib-0062] Rajagopalan, S. , Park, B. , Palanivel, R. , Vinayachandran, V. , Deiuliis, J. A. , Gangwar, R. S. , Das, L. , Yin, J. , Choi, Y. , Al‐Kindi, S. , Jain, M. K. , Hansen, K. D. , & Biswal, S. (2020). Metabolic effects of air pollution exposure and reversibility. The Journal of Clinical Investigation, 130, 6034–6040. 32780721 10.1172/JCI137315 PMC7598058

[phy270536-bib-0063] Rajagopalan, S. , Vergara‐Martel, A. , Zhong, J. , Khraishah, H. , Kosiborod, M. , Neeland, I. J. , Dazard, J. E. , Chen, Z. , Munzel, T. , Brook, R. D. , Nieuwenhuijsen, M. , Hovmand, P. , & Al‐Kindi, S. (2024). The urban environment and cardiometabolic health. Circulation, 149, 1298–1314. 38620080 10.1161/CIRCULATIONAHA.123.067461 PMC11093754

[phy270536-bib-0064] Reinhard, C. , Eder, G. , Fuchs, H. , Ziesenis, A. , Heyder, J. , & Schulz, H. (2002). Inbred strain variation in lung function. Mammalian Genome, 13, 429–437. 12226708 10.1007/s00335-002-3005-6

[phy270536-bib-0065] Ribble, A. , Hellmann, J. , Conklin, D. J. , Bhatnagar, A. , & Haberzettl, P. (2023). Fine particulate matter (PM(2.5))‐induced pulmonary oxidative stress contributes to increases in glucose intolerance and insulin resistance in a mouse model of circadian dyssynchrony. The Science of the Total Environment, 877, 162934. 36934930 10.1016/j.scitotenv.2023.162934 PMC10164116

[phy270536-bib-0066] Sagheer, U. , Al‐Kindi, S. , Abohashem, S. , Phillips, C. T. , Rana, J. S. , Bhatnagar, A. , Gulati, M. , Rajagopalan, S. , & Kalra, D. K. (2024). Environmental pollution and cardiovascular disease: Part 1 of 2: Air pollution. JACC Adv, 3, 100805. 38939391 10.1016/j.jacadv.2023.100805 PMC11198409

[phy270536-bib-0067] Schulz, H. , Johner, C. , Eder, G. , Ziesenis, A. , Reitmeier, P. , Heyder, J. , & Balling, R. (2002). Respiratory mechanics in mice: Strain and sex specific differences. Acta Physiologica Scandinavica, 174, 367–375. 11942924 10.1046/j.1365-201x.2002.00955.x

[phy270536-bib-0068] Singh, P. , O'Toole, T. E. , Conklin, D. J. , Hill, B. G. , & Haberzettl, P. (2021). Endothelial progenitor cells as critical mediators of environmental air pollution‐induced cardiovascular toxicity. American Journal of Physiology. Heart and Circulatory Physiology, 320, H1440–H1455. 33606580 10.1152/ajpheart.00804.2020 PMC8260385

[phy270536-bib-0069] Stucki, L. , Betner, S. , Selander, J. , Lohmus, M. , Akesson, A. , & Eriksson, C. (2023). Sociodemographic inequalities in long‐term exposure to air pollution, road traffic noise, and greenness: A population‐based cohort study of women. Environmental Epidemiology, 7, e279. 38912394 10.1097/EE9.0000000000000279 PMC11189682

[phy270536-bib-0070] Su, C. , Breitner, S. , Schneider, A. , Liu, L. , Franck, U. , Peters, A. , & Pan, X. (2016). Short‐term effects of fine particulate air pollution on cardiovascular hospital emergency room visits: A time‐series study in Beijing, China. International Archives of Occupational and Environmental Health, 89, 641–657. 26547916 10.1007/s00420-015-1102-6

[phy270536-bib-0071] Sun, Q. , Yue, P. , Deiuliis, J. A. , Lumeng, C. N. , Kampfrath, T. , Mikolaj, M. B. , Cai, Y. , Ostrowski, M. C. , Lu, B. , Parthasarathy, S. , Brook, R. D. , Moffatt‐Bruce, S. D. , Chen, L. C. , & Rajagopalan, S. (2009). Ambient air pollution exaggerates adipose inflammation and insulin resistance in a mouse model of diet‐induced obesity. Circulation, 119, 538–546. 19153269 10.1161/CIRCULATIONAHA.108.799015 PMC3845676

[phy270536-bib-0072] Tan, J. T. M. , Cheney, C. V. , Bamhare, N. E. S. , Hossin, T. , Bilu, C. , Sandeman, L. , Nankivell, V. A. , Solly, E. L. , Kronfeld‐Schor, N. , & Bursill, C. A. (2024). Female Psammomys obesus are protected from circadian disruption‐induced glucose intolerance, cardiac fibrosis and adipocyte dysfunction. International Journal of Molecular Sciences, 25, 7265. 39000372 10.3390/ijms25137265 PMC11242371

[phy270536-bib-0073] Tiberi, J. , Cesarini, V. , Stefanelli, R. , Canterini, S. , Fiorenza, M. T. , & La Rosa, P. (2023). Sex differences in antioxidant defence and the regulation of redox homeostasis in physiology and pathology. Mechanisms of Ageing and Development, 211, 111802. 36958540 10.1016/j.mad.2023.111802

[phy270536-bib-0074] Titchenell, P. M. , Lazar, M. A. , & Birnbaum, M. J. (2017). Unraveling the regulation of hepatic metabolism by insulin. Trends in Endocrinology and Metabolism: TEM, 28, 497–505. 28416361 10.1016/j.tem.2017.03.003 PMC5477655

[phy270536-bib-0075] Wang, M. , Hou, Z. H. , Xu, H. , Liu, Y. , Budoff, M. J. , Szpiro, A. A. , Kaufman, J. D. , Vedal, S. , & Lu, B. (2019). Association of Estimated Long‐term Exposure to air pollution and traffic proximity with a marker for coronary atherosclerosis in a Nationwide study in China. JAMA Network Open, 2, e196553. 31251382 10.1001/jamanetworkopen.2019.6553 PMC6604100

[phy270536-bib-0076] Wang, Y. , Xiong, L. , Huang, X. , Ma, Y. , Zou, L. , Liang, Y. , Xie, W. , Wu, Y. , Chang, X. , Wang, Z. , & Tang, M. (2022). Intermittent exposure to airborne particulate matter induces subcellular dysfunction and aortic cell damage in BALB/c mice through multi‐endpoint assessment at environmentally relevant concentrations. Journal of Hazardous Materials, 424, 127169. 34592597 10.1016/j.jhazmat.2021.127169

[phy270536-bib-0077] Wang, Y. Y. , Li, Q. , Guo, Y. , Zhou, H. , Wang, Q. M. , Shen, H. P. , Zhang, Y. P. , Yan, D. H. , Li, S. , Chen, G. , Zhou, S. , He, Y. , Yang, Y. , Peng, Z. Q. , Wang, H. J. , & Ma, X. (2020). Long‐term exposure to airborne particulate matter of 1 mum or less and blood pressure in healthy young adults: A national study with 1.2 million pregnancy planners. Environmental Research, 184, 109113. 32199315 10.1016/j.envres.2020.109113

[phy270536-bib-0078] WHO . (2021). What are the WHO air quality guidelines. https://www.who.int/news‐room/feature‐stories/detail/what‐are‐the‐who‐air‐quality‐guidelines

[phy270536-bib-0079] WHO . (2024). Ambient (outdoor) air pollution. https://www.who.int/news‐room/fact‐sheets/detail/ambient‐(outdoor)‐air‐quality‐and‐health

[phy270536-bib-0080] Xie, X. , Wang, Y. , Yang, Y. , Xu, J. , Zhang, Y. , Tang, W. , Guo, T. , Wang, Q. , Shen, H. , Zhang, Y. , Yan, D. , Peng, Z. , Chen, Y. , He, Y. , & Ma, X. (2018). Long‐term effects of ambient particulate matter (with an aerodynamic diameter </=2.5 mum) on hypertension and blood pressure and attributable risk among reproductive‐age adults in China. Journal of the American Heart Association, 7(9), e008553. 29700042 10.1161/JAHA.118.008553 PMC6015291

[phy270536-bib-0081] Yan, R. , Ku, T. , Yue, H. , Li, G. , & Sang, N. (2020). PM(2.5) exposure induces age‐dependent hepatic lipid metabolism disorder in female mice. Journal of Environmental Sciences (China), 89, 227–237. 31892394 10.1016/j.jes.2019.10.014

[phy270536-bib-0082] Zhong, L. X. , Li, X. N. , Yang, G. Y. , Zhang, X. , Li, W. X. , Zhang, Q. Q. , Pan, H. X. , Zhang, H. H. , Zhou, M. Y. , Wang, Y. D. , Zhang, W. W. , Hu, Q. S. , Zhu, W. , & Zhang, B. (2019). Circadian misalignment alters insulin sensitivity during the light phase and shifts glucose tolerance rhythms in female mice. PLoS One, 14, e0225813. 31851682 10.1371/journal.pone.0225813 PMC6919582

[phy270536-bib-0083] Zhu, L. , Zou, F. , Yang, Y. , Xu, P. , Saito, K. , Othrell Hinton, A., Jr. , Yan, X. , Ding, H. , Wu, Q. , Fukuda, M. , Sun, Z. , Tong, Q. , & Xu, Y. (2015). Estrogens prevent metabolic dysfunctions induced by circadian disruptions in female mice. Endocrinology, 156, 2114–2123. 25807042 10.1210/en.2014-1922 PMC4430614

[phy270536-bib-0084] Zimmet, P. , Alberti, K. , Stern, N. , Bilu, C. , El‐Osta, A. , Einat, H. , & Kronfeld‐Schor, N. (2019). The circadian syndrome: Is the metabolic syndrome and much more! Journal of Internal Medicine, 286, 181–191. 31081577 10.1111/joim.12924 PMC6851668

